# An 11-bp Indel Polymorphism within the *CSN1S1* Gene Is Associated with Milk Performance and Body Measurement Traits in Chinese Goats

**DOI:** 10.3390/ani9121114

**Published:** 2019-12-11

**Authors:** Yanghai Zhang, Ke Wang, Jinwang Liu, Haijing Zhu, Lei Qu, Hong Chen, Xianyong Lan, Chuanying Pan, Xiaoyue Song

**Affiliations:** 1 Key Laboratory of Animal Genetics, Breeding and Reproduction of Shaanxi Province, College of Animal Science and Technology, Northwest A&F University, Yangling, Shaanxi 712100, China; yhzhang1997@163.com (Y.Z.); lp_wangke@163.com (K.W.); chenhong1212@126.com (H.C.); lanxianyong79@126.com (X.L.); 2 Shaanxi Provincial Engineering and Technology Research Center of Cashmere Goats, Yulin University, Yulin, Shaanxi 719000, China; ljw_yl@163.com (J.L.); haijingzhu@yulinu.edu.cn (H.Z.); ylqulei@126.com (L.Q.); 3 Life Science Research Center, Yulin University, Yulin, Shaanxi 719000, China

**Keywords:** goat, casein alpha s1, insertion/deletion, milk performance, body measurement traits

## Abstract

**Simple Summary:**

In the field of animal breeding, selection using molecular genetic markers, such as insertions/deletions (indels), is a novel method to reduce the generation interval and more accurately improve performance. The casein alpha s1 (*CSN1S1*) gene is one of the major genes regulating the milk performance of mammals, and it is also relevant to body development. Previous studies have shown that an 11-bp indel in this gene was strongly associated with goat litter size, however, its effects on milk performance and body measurement traits have not been studied. In this study, this indel was genotyped in three Chinese goat breeds, and further analysis results revealed that this indel polymorphism was significantly associated with milk performance in dairy goats, as well as affecting the body measurement traits in all three breeds. These findings hint that the 11-bp indel within *CSN1S1* gene could be utilized for effectively selecting goats with excellent performances in terms of production and breeding.

**Abstract:**

The casein alpha s1 (*CSN1S1*) gene encodes α-s1 casein, one of the proteins constituting milk, which affects milk performance, as well as improving the absorption of calcium and bone development in mammals. A previous study found that an 11-bp insertion/deletion (indel) of this gene strongly affected litter size in goats. However, to our knowledge, the relationships between this polymorphism and the milk performance and body measurement traits of goats have not been reported. In this paper, the previously identified indel has been recognized in three Chinese goat breeds, namely the Guanzhong dairy goat (GZDG; n = 235), Shaanbei white cashmere goat (SBWC; n = 1092), and Hainan black goat (HNBG; n = 278), and the following three genotypes have been studied for all of the breeds: insertion/insertion (II), deletion/deletion (DD), and insertion/deletion (ID). The allele frequencies analyzed signified that the frequencies of the “D” allele were higher (47.8%–65.5%), similar to the previous report, which indicates that this polymorphism is genetically stable in different goat breeds. Further analysis showed that this indel was markedly associated with milk fat content, total solids content, solids-not-fat content, freezing point depression, and acidity in GZDG (*p* < 0.05), and also affected different body measurement traits in all three breeds (*p* < 0.05). The goats with II genotypes had superior milk performance, compared with the others; however, goats with DD genotypes had better body measurement sizes. Hence, it may be necessary to select goats with an II or DD genotype, based on the desired traits, while breeding. Our study provides information on the potential impact of the 11-bp indel polymorphism of the *CSN1S1* gene for improving the milk performance and body measurement traits in goats.

## 1. Introduction

The goat industry is one of the oldest animal husbandry industries, with a high potential; however, the actual efficiency of goat breeding is not high, and, at present, the demand for goat products exceeds the supply [1,2,3,4,5]. Thus, a pressing question that has been proposed is how to improve the production performance of goats [6,7]. Traditional breeding, for example, using estimated breeding values (EBVs), has been extremely successful in observing the genetic progress. However, the aim of breeding programs is to predict the genetic merit of the next generation and to improve the genetic performance [8]. Based on the identification of genetic DNA markers, such as single nucleotide polymorphisms (SNPs) and insertions/deletions (Indels), novel breeding methods were built, including marker-assisted selection (MAS) and genomic selection (GS), which would significantly reduce the generation interval and increase accuracy [8,9]. At present, some candidate genes and DNA markers have been identified for improving reproductive and body measurement traits in livestock [10,11,12,13], but more crucial and powerful candidate genes require identification in order to comprehensively improve production traits in goat breeding.

Casein gene family (CSNs) is linked as a *CSN1S1–CSN2–CSN1S2–CSN3* gene cluster encoding the following four caseins: αs1, β, αs2, and κ. Milk proteins are constituted of whey proteins and above-mentioned four kinds of caseins, where caseins account for the majority of the milk [14]. Among these casein genes, the casein alpha s1 (*CSN1S1*) gene encodes α-s1 casein, which is mainly expressed in breast tissues, especially in the lactating breasts of females (https://www.proteinatlas.org/). This type of casein is also the most abundant casein in bovine and caprine milk, and can provide sufficient amino acids for animal growth and development [15]. Therefore, this gene exerts a crucial role in milk yield and composition. In previous studies, the goat *CSN1S1* gene has shown at least 18 alleles, including A, B1, B2, B3, B4, B′, C, D, E, F, G, H, I, L, M, N, O1, and O2 [16,17,18], and many studies have shown that these polymorphisms affect caprine milk performance in different goat breeds. The N and F alleles of *CSN1S1* have been shown to increase the milk yield in the Xinong Saanen dairy goat [19]; the BB and AB genotypes have been associated with the highest milk protein and fat in Sarda goats [20]; and some polymorphisms have been shown to affect the fat content, protein content, and coagulation properties of goat milk [21,22]. In other ruminants, such as cows [23], sheep [24], and buffalo [25], *CSN1S1* polymorphisms have also been shown to significantly affect the milk production traits.

On the other hand, casein has been considered to have a high nutritional value, with the potential to improve bone development and growth (as it contains almost all of the necessary amino acids), as well as increasing the absorption of calcium and other micro-elements in the infant gut by complexing and solubilizing calcium ions [14,15]. Animal experiments in vivo have convincingly testified that casein or casein phosphopeptides (CPPs; originating from the digestion of casein) can promote calcium uptake and bone formation, as well as increase bone strength and quality, and body weight [26,27,28]. Therefore, this gene may play a crucial role in the body size, structure, and development of goats.

Furthermore, *CSN1S1* may even play an important role, in terms of reproductive traits. The *BMPR1B* gene (also called *Fec^B^*) is a famous reproduction-related gene, as it can significantly regulate the ovulation rate in sheep [29,30]. As far back as 1994, Montgomery et al. determined that the *Fec^B^* gene is located on chromosome six in sheep, and identified genetic linkages with platelet-derived growth factor receptor-alpha (*PDGFRA*), *CSN1S1,* and other casein genes [31]. According to the current genome sequence, the number of bases between the *BMPR1B,* and *PDGFRA* and *CSN1S1* that differ by 42.7 and 60.5 Mb in sheep (NC_040257.1), respectively; these are 40.0 and 55.6 Mb in goat (NC_030813.1), respectively. This information builds the basis for the conclusions from 1994 [31]. Furthermore, Lan et al. found that the polymorphisms in other casein family genes (*CSN3* and *CSN1S2*) had significant positive effects on the litter size of the Xinong Saanen dairy goat [32]. Therefore, we speculated that the *CSN1S1* gene may be linked to the *BMPR1B* gene in goats, and thus, the variations within *CSN1S1* might affect the regulation of fecundity [1].

As a powerful candidate gene in livestock breeding, our previous study found that an 11-bp indel within the *CSN1S1* gene strongly affected the litter size in a larger sample of the Shaanbei white cashmere goat (SBWC) [1]. However, the relationships between this polymorphism and the milk performance and body measurement traits of goats have not yet been studied, to our knowledge. Therefore, based on our preliminary work, this study is aimed at unveiling the association of the 11-bp indel of *CSN1S1,* and the milk and body measurement traits in Chinese goats, which would contribute to selecting a herd with a high productivity using the MAS method in goat breeding.

## 2. Materials and Methods

### 2.1. Sample and Data Collection

All of the animal experiments in this study conformed to the relevant laws and guidelines and were approved by the Faculty of Animal Policy and Welfare Committee of Northwest A&F University (NWAFAC1008).

A total of 1605 ear samples (small ear notches) were randomly collected from the following three Chinese indigenous goat breeds: Guanzhong dairy goats (GZDG; n = 235), Shaanbei white cashmere goats (SBWC; n = 1092), and Hainan black goats (HNBG; n = 278). The dairy-use breed GZDG were adult healthy female goats (2–4 years old) from a local farm in Sanyuan county of Shaanxi Province [33]. These goats were unrelated, and the milk performance indices were recorded by Lan et al. in 2013 [33]. Based on a 305-day lactation period, different lactation data were collected from the GZDG [33]. The milk samples were collected twice a day (05:00 and 17:00) when the dairy goats were milked. Then, the milk samples were measured for the following eight milk performance indices: milk fat content (%), protein content (%), total solids (TS) content (%), solids-not-fat (SNF) content (%), lactose content (%), density, freezing point depression (FPD; °C), and acidity (pH) [33]. Each milk performance index was taken as the average of two values (05:00 and 17:00). Additionally, the body measurement traits of the GZDG were collected, including the body height, body length, height at hip cross, chest circumference, chest width, hip width, and cannon circumference.

The meat and cashmere dual-purpose SBWC goats were adult females (2–3 years old), and reared in the Shaanbei white cashmere goat breeding farm in Yulin city of Shaanxi Province [1,34]. Ear samples were randomly collected in these populations between 2016 and 2018 [1,34]. These goats were unrelated and healthy. For this breed, we measured seven values of body size, including the body height, body length, height at hip cross, chest circumference, chest depth, chest width, and cannon circumference [34]. In addition, the adult female HNBGs (2–3 years old) were raised at a local farm in Zanzhou country of Hainan Province [35]. This breed is a famous meat-type of goat breed; therefore, the data of the body measurement traits were recorded, including the body height, body length, body weight, chest circumference, chest depth, chest width, hip width, and cannon circumference [35].

### 2.2. DNA Extraction and Indel Genotyping

The genomic DNA samples were extracted from the ear tissue samples using the high-salt extraction method [36]. A Nanodrop 1000 Spectrometer (Thermo Scientific, Waltham, MA, USA) was used to detect the DNA purity and quality. Then, the extracted DNA were diluted to 10 ng/μL and stored at −20 °C.

According to the previous report [1], a pair of primers (F: 5′-GCTGGAAGCAGTTCGTCA-3′; R: 5′-GGGTTGATAGCCTTGTATGTT-3′) was synthesized to detect the 11-bp indel within the *CSN1S1* gene in all of the goats. The PCR amplification processes used were the same as in the previous study, including the primers, PCR reaction mixture, and amplification condition. If the PCR products migrated at the same position on the agarose gel when they were genotyped by 3.0% agarose gel electrophoresis, as previously reported, the genotype of each goat could be visualized. For example, one band appearing at 170 bp meant that the individual was of a homozygote insertion type (insertion/insertion, II), one band appearing at 159 bp meant that the individual was of a homozygote deletion type (deletion/deletion, DD), and two bands appearing in a lane meant that the individual was of a heterozygote type (insertion/deletion, ID). Meanwhile, the PCR products were confirmed by sequencing and compared to the reference genome (NC_030813.1; Tsingke company, Xi’an, Shaanxi, China).

### 2.3. Statistical Analysis

The genotypes, allele frequencies, and a test for the Hardy–Weinberg equilibrium (HWE) were calculated using the SHEsis platform (http://analysis.bio-x.cn/myAnalysis.php) [37]. The population indices (polymorphism information content, PIC; homozygosity, Ho; heterozygosity, He; effective allele numbers, Ne) were calculated following Nei’s methods [38].

The general linear model (GLM) of the analysis of variance (ANOVA) was used to analyze the relationships between the different genotypes and economically important traits using the SPSS 23.0 statistical software package (IBM, New York, NY, USA). The statistical model for the milk performance traits used was as follows: Y_ijk_ = µ + L_i_ + G_j_ + (LG)_ij_ + ε_ijk_, where Y_ijk_ is the observation of the milk performance indices, µ is the overall mean of each index, L_i_ is the effect due to i_th_ lactation, G_j_ is the fixed effect of j_th_ genotype, (LG)_ij_ is interaction between the i_th_ lactation and the j_th_ genotype, and ε_ijk_ is the random error [33]. Meanwhile, for the body measurement traits, the GLM was calculated as follows: Y_il_ = μ + A_i_ + G_l_ + ε_il_, where Y_il_ represents the observations of the body measurement traits, μ is the population mean, A_i_ is the fixed effect of age, G_l_ is the fixed effect of genotype, and ε_il_ is the random error [39,40]. The model excluded the effects of farm, sex, and season of birth, which had no significant effects on the variation of traits in this population [40]. The different breeds were raised in their respective farms and analyzed separately. All of the data were presented as the mean ± standard error (S.E.), and *p* < 0.05 was considered to be significant.

## 3. Results

### 3.1. Genetic Parameter of 11-bp Indel of CSN1S1 in Three Chinese Goat Breeds

**Figure 1:**
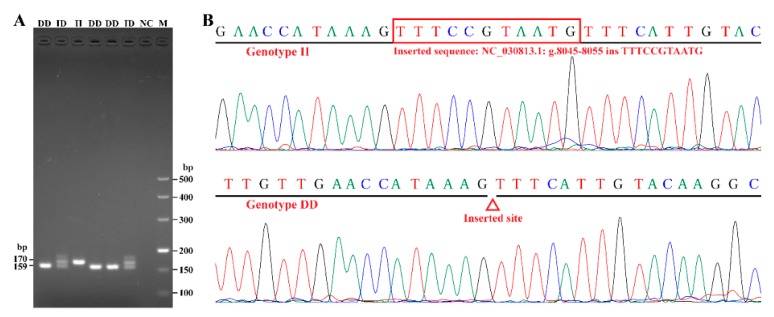
Visualization and sequencing of the 11-bp indel under study in the goat casein alpha s1 (*CSN1S1*) gene. (**A**) The electrophoresis pattern of the 11-bp indel within the goat *CSN1S1* gene. Lanes 1, 4, and 5 show the homozygote deletion type (DD); lane 3 shows the homozygote insertion type (II); lanes 2 and 6 show the heterozygote type (ID); lane 7 is a negative control; and lane 8 is a DNA ladder marker. (**B**) The sequence chromatograms of the 11-bp indel in goat *CSN1S1*. The upper and lower panels show the sequence chromatograms of the II and DD genotypes, respectively.

Based on the gel electrophoresis pattern and sequence chromatograms (Figure 1), an 11-bp indel of the *CSN1S1* gene was detected in all of the tested goats. The polymorphism was present in the following three variants: the homozygote insertion type (II, 170 bp), the homozygote deletion type (DD, 159 bp), and the heterozygote type (ID, 170 bp and 159 bp; Figure 1A). After sequence alignment with the updated reference genome (NC_030813.1), it was found that the polymorphism was an insertion mutation, and the position changed from 6654–6664 to 8045–8055 (Figure 1B). Hence, we redefined this polymorphism as NC_030813.1: g.8045–8055insTTTCCGTAATG, compared to the previous name (NC_030813.1: g.6654–6664delTTTCCGTAATG) given by Wang et al. [1]. Moreover, in this study, the heterozygote type “ID” actually showed three bands on the agarose gel (Figure 1A), as the top band was a heteroduplex [41,42].

Subsequently, the genotype and allele frequencies of this polymorphism in the three goat breeds were calculated (Table 1). The minor allele frequencies of this indel in GZDG, SBWC, and HNBG were 0.345, 0.478, and 0.448, respectively. The frequency of the ID genotype was higher than the other genotypes in SBWC and HNBG; however, in the GZDG population, the highest genotype frequency (0.481) was the homozygote deletion type “DD”. Based on the frequency numbers of the different genotypes, the population indices (Ho, He, Ne, and PIC), indicating the degrees of genetic variation, were calculated (Table 1). The Ho, He, and Ne values of this polymorphism were high in all three populations; meanwhile, the PIC value showed that all of the breeds had moderate genetic diversity (0.25 < PIC < 0.5) at this indel locus of the *CSN1S1* gene. In addition, the χ^2^ test results demonstrated that no breed was in accordance with HWE (*p* < 0.05; Table 1).

### 3.2. Association between the 11-bp Indel and Milk Performance Traits in the Guanzhong Dairy Goat

To assess the effect of the 11-bp indel on the milk performance traits, the milk samples were collected and eight milk physicochemical indices were measured in the Guanzhong dairy goat (GZDG) [33]. The analysis result demonstrated that this indel was significantly associated with five of the indices in dairy goat, including milk fat content (%), TS content (%), SNF content (%), FPD (°C), and acidity (pH; Table 2; *p* < 0.05). Goats with II or ID genotypes had higher values of milk physicochemical indices compared with individuals with the DD genotype (*p* < 0.05).

### 3.3. Association Analysis of the 11-bp Indel with Body Measurement Traits in Three Goat Breeds

The body measurement traits can directly reflect the skeleton structure, body size, and growth development, which are related to the physiological function and the production performance of goats [35]. Here, the various body sizes were collected to analyze the relationships between this polymorphism and the body measurement traits in different breeds, including the meat-use breeds (HNBG and SBWC) and the dairy-use breed (GZDG). The association analysis results revealed that the 11-bp indel was significantly associated with different body measurement traits in different goat breeds (Table 3). The indel was significantly related to the BH of all three of the breeds (*p* < 0.05). Two body measurement traits—body height and body length—were related in the GZDG population (*p* < 0.05); six kinds of body measurement traits—body height, body length, height at hip cross, chest circumference, chest width, and cannon circumference—were associated with this polymorphism in SBWC goats (*p* < 0.05); and, in the HNBG population, three different body measurement traits—body height, chest depth, and body weight—were associated with the polymorphism (*p* < 0.05). An interesting tendency that was observed was that female goats with DD or ID genotypes had greater body measurement traits than those of other genotypes in the three goat breeds (*p* < 0.05).

## 4. Discussion

The *CSN1S1* gene encodes α-s1 casein, one of the proteins constituting milk that can improve calcium uptake in mammals, which affects milk performance and growth development [14,15,23,26]. Many studies have shown that *CSN1S1* polymorphisms can affect the milk performance in different goat breeds [19,20,21,22]. Our team has previously identified an 11-bp indel within the *CSN1S1* gene, which strongly affected litter size in a large sample of SBWC goats (n = 3047) [1]. However, whether this polymorphism affects the milk performance and body measurement traits of goats has not yet been investigated. In this paper, we detected this indel in three local goat breeds, and showed the following three genotypes: II (homozygote insertion type), ID (heterozygote type), and DD (homozygote deletion type). As the reference caprine genome sequence of *CSN1S1* (NC_030813.1) was updated, the polymorphism was redefined as NC_030813.1: g.8045–8055insTTTCCGTAATG. In three goat breeds, the average frequency of the “D” allele was 56.2%, which was similar to that reported in a previous report on SBWC goats (54.0%) by Wang et al. [1]. However, in SBWC goats, the frequency of the “D” allele was lower than that of the previous study, which could be due to the sample sizes in this experiment being smaller. Furthermore, no breed was in accordance with the HWE, suggesting that the populations of the three breeds were not in dynamic equilibrium, due to migration, genetic drift, and artificial selection [43]. Overall, this polymorphic mutation had a high frequency and was present in all three goat breeds from different regions, indicating that this polymorphic mutation is genetically stable in goats and shows potential as a candidate DNA marker in molecular breeding progress.

Subsequently, the association analysis uncovered that the strong influences of the 11-bp indel of *CSN1S1* on the milk performance traits in dairy goats (Table 2). According to the results, dairy goats of the II or ID genotypes demonstrated better milk performance traits, particularly in terms of milk protein content (%), TS content (%), and SNF content (%; *p* < 0.05). Higher values of these traits indicate that a goat’s milk contains more dry matter, especially proteins, because there is no difference in milk fat content (%) and lactose content (%) among individuals of different genotypes (*p* > 0.05). The reason for this difference may be due to the fact that the *CSN1S1* gene encodes α-s1 casein, which is the richest protein in milk [14]. Therefore, the polymorphism may affect the expression of the protein, resulting in changes in milk protein content and other components of dairy goats’ milk. The increase in the milk protein improves the nutritional value of milk and is also beneficial in the cheese-making process [44,45]. However, this may also have some negative effects. Some literature indicates that the variants led to increased milk components, but reduced the milk yield [46,47,48]. Regrettably, in this study, because of the lack of data on the milk yield of dairy goats, the effect of this indel on this aspect cannot be studied. In the future, more comprehensive data about milk performance will need to be collected in order to explore the impact of this 11-bp indel.

In addition, as caseins could promote bone formation and body development by increasing intestinal calcium absorption and bioavailability [14,26,27,28,49], the effect of this 11-bp indel of the *CSN1S1* gene on the body measurement traits of goats is worth studying. The association analysis results demonstrated that this polymorphism significantly affected the body measurement traits of goats and affected the different body measurement sizes in different goat breeds (Table 3; *p* < 0.05). These differences might be due to the diversity of goat breeds, and individuals with DD or ID genotypes had better body measurement traits, compared with the II type, in all three breeds (*p* < 0.05). Therefore, for selecting the body measurement trait of goats, the DD genotype of the 11-bp indel within the *CSN1S1* gene is the dominant type.

Intriguingly, the dominant genotype of milk performance (Table 2) and litter size [1] in goats was the II genotype for the 11-bp indel polymorphism within the *CSN1S1* gene, but the dominant genotype of the body measurement trait is the DD genotype and individuals with the II genotype had lower body measurement sizes (Table 3). In general, female animals, after parturition and during lactation, need to provide more nutrients to their offspring, and so their body conditions decline [50,51,52]. For goat breeds dedicated to milk production, their body sizes are not as big as that of meat-producing goats, because it is less important to increase body size instead of milk production. This may explain why the dominant genotypes are the opposite in the two traits of goats. Therefore, according to the existing analysis results, the genotype selection for breeding can be carried out based on the use of goats, as follows: if the milk performance needs to be improved, for example, in dairy goat breeds, goats with the II genotype of the 11-bp indel in the *CSN1S1* gene should be selected; on the other hand, for improving body measurement traits in goats, such as in meat-use or meat–cashmere dual purpose goat breeds, the DD genotype should be chosen. However, as the number of samples used to study milk performance traits in this experiment was not large and only one breed type was used (only GZDG), no decisive conclusion can be drawn. It is necessary to increase the sample size and collect more production performance indices with different goat breeds for further conclusions to be drawn.

In addition, considering that this indel is an intron variation and often has no direct effect on gene expression, it might be linked with other polymorphisms in the exon or the transcriptional regulation region to exert indirect effects [53,54]. In fact, in our previous study on litter size, we explored whether this 11-bp indel was linked to the polymorphisms of the *BMPR1B* gene in goats; however, the Q279R mutation of *BMPR1B* was not found in SBWC population [1]. Therefore, it is possible that this polymorphism affects litter size by it linking to other potential mutations of the *BMPR1B* gene or other genes in goats [1]. Unfortunately, at present, most studies have only focused on the relationships between this gene and the milk performance traits, and only one study has reported on the associations between genetic variants of the *CNS1S1* gene and different economically important traits in goats [55]. The author found that some SNPs within exons 9–11 significantly affected the milk yield, litter size, and body measurement traits in the Xinong Sannen dairy goats [55]. Therefore, we suspect that this indel might link with these SNPs to control the production traits of goats. In addition, the intronic polymorphisms might also change the transcription and translation processes of the gene by binding trans-acting factors [56,57], altering the gene splicing pattern [58,59], or mediating the biogenesis of the microRNA [60]. The possible mechanisms of this 11-bp indel polymorphism (i.e., those affecting the economically important traits) still need to be explored.

## 5. Conclusions

An 11-bp indel in the *CSN1S1* gene significantly affected the milk performance and body measurement traits in goats, and the dominant genotypes were opposite in the two traits. Therefore, the genotype selection of breeding in the MAS program can be carried out based on the use of goats, as follows: if the milk performance needs to be improved, for example, in dairy goat breeds, goats with the II genotype of this polymorphism should be selected; on the other hand, for improving the body measurement traits into goats, such as in meat-use breeds, the DD genotype should be chosen.

## Figures and Tables

**Table 1:** The genotype and allele frequencies and population indices of the 11-bp indel of *CSN1SI* gene in three different goat breeds.

Breeds	Total Sizes	Genotype Frequencies (N)			Allele Frequencies		Population Parameters				χ^2^ (*p* Value)
		II	ID	DD	I	D	Ho	He	Ne	PIC	
GZDG	235	0.170 (40)	0.429 (82)	0.481 (113)	0.345	0.655	0.548	0.452	1.824	0.350	12.173 (*p* = 4.85 × 10^−4^)
SBWC	1092	0.295 (322)	0.454 (496)	0.251 (274)	0.522	0.478	0.501	0.499	1.996	0.375	8.809 (*p* = 0.003)
HNBG	278	0.255 (71)	0.385 (107)	0.360 (100)	0.448	0.552	0.505	0.495	1.978	0.372	13.670 (*p* = 2.18 × 10^−4^)

Note: GZDG—Guanzhong dairy goat; SBWC—Shaanbei white cashmere goat; HNBG—Hainan black goat; Ho—homozygosity; He—heterozygosity; Ne—effective allele numbers; PIC—polymorphism information content.

**Table 2:** Association between the indel in the *CSN1S1* gene and milk performance indices in the Guanzhong dairy goats.

Milk Performance Indices	Genotypes (N)			*p* Value
	II (11)	ID (35)	DD (46)	
Fat content (%)	4.61 ± 0.40	4.36 ± 0.20	3.86 ± 0.15	0.103
Protein content (%)	3.93 ^a^ ± 0.21	3.73 ^ab^ ± 0.12	3.38 ^b^ ± 0.07	0.006
TS content (%)	13.73 ^a^ ± 0.57	13.38 ^ab^ ± 0.34	12.44 ^b^ ± 0.21	0.018
SNF content (%)	8.99 ^a^ ± 0.19	8.91 ^a^ ± 0.13	8.46 ^b^ ± 0.08	0.003
Lactose content (%)	3.88 ± 0.09	4.02 ± 0.08	3.95 ± 0.04	0.481
Density	1028.80 ± 0.45	1029.25 ± 0.46	1028.27 ± 0.27	0.144
FPD (°C)	0.50^a^ ± 0.01	0.50 ^a^ ± 0.01	0.48 ^b^ ± 0.01	0.043
Acidity (pH)	7.49^a^ ± 0.51	6.79 ^ab^ ± 0.33	6.22 ^b^ ± 0.19	0.046

Note: TS—total solids; SNF—solids-not-fat; FPD—freezing point depression. Values with different letters (a and b) within the same row differ significantly at *p* < 0.05.

**Table 3:** Relationships between the 11-bp indel of the *CSN1S1* gene and body measurement traits in the three goat breeds.

Breeds	Body Measurement Traits	Genotypes (N)			*p* Value
		**II (11**)	**ID (35)**	**DD (46)**	
GZDG	Body height (cm)	67.98 ^c^ ± 3.09	69.05 ^b^ ± 2.89	71.26 ^a^ ± 0.90	0.009
	Body length (cm)	75.54 ^b^ ± 0.98	77.24 ^a^ ± 1.18	77.60 ^a^ ± 0.68	0.043
	Height at hip cross (cm)	68.98 ± 2.91	69.33 ± 1.11	70.81 ± 0.95	0.571
	Chest circumference (cm)	89.18 ± 0.94	88.28 ± 0.98	88.92 ± 0.82	0.841
	Chest width (cm)	28.70 ± 0.85	27.81 ± 0.55	29.17 ± 0.49	0.180
	Hip width (cm)	21.94 ± 1.41	23.54 ± 0.59	22.26 ± 0.67	0.293
	Cannon circumference (cm)	11.34 ± 0.40	10.89 ± 0.21	10.30 ± 0.27	0.099
		**II (247)**	**ID (380)**	**DD (195)**	
SBWC	Body height (cm)	56.62 ^c^ ± 0.29	58.11 ^b^ ± 0.19	60.12 ^a^ ± 0.33	1.61 × 10^−16^
	Body length (cm)	66.42 ^b^ ± 0.31	67.95 ^a^ ±0.23	68.62 ^a^ ± 0.33	1.23 × 10^−6^
	Height at hip cross (cm)	59.41 ^c^ ± 0.29	60.66 ^b^ ± 0.21	61.97 ^a^ ± 0.30	4.22 × 10^−9^
	Chest circumference (cm)	84.97 ^b^ ± 0.49	86.78 ^a^ ± 0.35	86.93 ^a^ ± 0.50	0.003
	Chest width (cm)	19.59 ^b^ ± 0.18	20.36 ^a^ ±0.14	20.48 ^a^ ±0.18	3.90 × 10^−4^
	Chest depth (cm)	30.61 ± 01.16	30.15 ± 0.19	30.46 ± 0.18	0.851
	Cannon circumference (cm)	8.13 ^b^ ± 0.05	8.32 ^a^ ± 0.03	8.26 ^a^ ± 0.05	0.003
		**II (26)**	**ID (39)**	**DD (36)**	
HNBG	Body height (cm)	51.50 ^b^ ± 0.89	53.63 ^a^ ± 0.64	53.98 ^a^ ± 0.65	0.049
	Body length (cm)	54.71 ± 1.14	56.50 ± 0.75	57.55 ± 0.64	0.069
	Body weight (kg)	26.79 ^b^ ± 1.48	29.35 ^ab^ ± 1.17	31.34 ^a^ ± 1.00	0.043
	Chest circumference (cm)	71.04 ± 1.39	73.00 ± 1.11	74.40 ± 0.90	0.137
	Chest width (cm)	15.10 ± 0.33	15.61 ± 0.33	15.51 ± 0.26	0.521
	Chest depth (cm)	25.83 ^b^ ± 0.47	26.67 ^ab^ ± 0.34	27.28 ^a^ ± 0.32	0.033
	Hip width (cm)	13.26 ± 0.35	13.51 ± 0.26	14.10 ± 0.17	0.069
	Cannon circumference (cm)	7.92 ± 0.13	8.08 ± 0.10	8.01 ± 0.09	0.621

Note: GZDG—Guanzhong dairy goat; SBWC—Shaanbei white cashmere goat; HNBG—Hainan black goat. Values with different letters (a and b) within the same row differ significantly at *p* < 0.05.
